# Explainability in the age of large language models for healthcare

**DOI:** 10.1038/s44172-025-00453-y

**Published:** 2025-07-17

**Authors:** Munib Mesinovic, Peter Watkinson, Tingting Zhu

**Affiliations:** 1 Department of Engineering Science, University of Oxford, Oxford, OX1 3PJ UK; 2 Nuffield Department of Clinical Neurosciences, University of Oxford, Oxford, OX3 9DU UK

**Keywords:** Mathematics and computing, Health care

## Abstract

Large language models show remarkable potential in healthcare but face critical explainability challenges that must be addressed before widespread clinical deployment. Here, Munib Mesinovic, Peter Watkinson and Tingting Zhu examine technical and regulatory solutions needed to develop trustworthy, transparent large language models for responsible healthcare integration.

## Teaser

Large language models show remarkable potential in healthcare but face critical explainability challenges that must be addressed before widespread clinical deployment. Here, we examine technical and regulatory solutions needed to develop trustworthy, transparent large language models for responsible healthcare integration.

## Introduction

Large language models (LLMs) have achieved impressive progress over the last few years, and, in healthcare, they can answer patients’ queries at an indistinguishable level from a clinician, predict patient trajectories using electronic health records, and write incredibly coherent medical administrative documents^1^. While these examples highlight the benefit of the technology, certain constraints must also apply. LLMs, like other AI models, must satisfy explainability constraints for stakeholders like clinicians and patients^2,3^. LLMs have unlocked the potential for bigger leaps forward in healthcare, but explaining them is another challenge entirely.

### The critical need for explainable AI in clinical settings

Explainability describes the property of an AI system to be transparent and interpretable, and its decisions to be evaluated for bias and fairness^4^. As^5^ suggests, no matter whether the perspective is legal, industrial, or economic, explainable AI cannot be separated from its requirements to be interpretable, trustworthy, accountable, and transparent. Definitions adopted from^4^ for these concepts describe:

- explainability “in the context of AI [as] the ability to understand and not just interpret the decisions or predictions made by an AI system”
- interpretability as the quality of “how well the output predictions, whether it be in clinical risk modelling or elsewhere, can be interpreted by behaviour in the input features”
- trust as the ability of the AI to “[satisfy] certain [contextual] confidence measures in prediction”
- And transparency as the “characteristic of an AI system to have its motivation, design, and implementation elucidated to stakeholders, whether it be through documentation or user-level testing or some other means”

These criteria gain even greater importance in sensitive domains, such as healthcare, where LLMs must be held to robust standards. Decisions about patients’ care affect people’s lives profoundly, and AI can often lead to unreliable results or biased decision-making^4,6^. For both clinicians and patients to understand and trust LLMs, they need to be able to “evaluate and identify how [their personal] data are being used and whether the outcome is correct”^4,6^. If LLMs are to revolutionise the healthcare sector and convince practitioners, explainability must be a requirement in addition to their impressive generative or predictive performance.

Beyond the necessity to build trust for trust’s sake, explainable LLMs in areas like healthcare could be a regulatory requirement. With the General Data Protection Regulation (GDPR) and national healthcare laws, there is rising agreement that even in cases where the output of models is only used as a recommendation or as support for clinicians’ and patients’ decisions, “clinicians must have a sufficient grasp of the medically-relevant factors involved in the model output”^7^. To that end, interpretability is no longer enough, and an array of explainability criteria like transparency and trustworthiness come into play if LLMs are to be used, ultimately, as a regulated and reliable component of a more efficient healthcare system^2,3^. Making LLMs satisfy these criteria, however, is not an easy task. Explainable approaches in the literature to interpreting machine learning models, like saliency maps^8^ or attribution methods^9^, fail here due to the complexity of LLMs^10^. LLMs’ generative and stochastic nature can cause the same input to generate a different output if we just run the same model again^10^. Additionally, LLMs’ in-context learning ability means their responses can change creatively and thus, explaining them requires catching up with this diverse reasoning^10^. The goalpost has changed. Where we previously aimed to explain AI models attempting to produce coherent sentences, we now aim to explain a foundation model with far greater ambitions.

### Technical approaches to enhancing LLM explainability

One strategy is to ask the model to explain its thinking, an approach called Chain-of-Thoughts (CoT) prompting. The explanations, however, are often not factually grounded and are susceptible to bias^11^. Another way is to construct more reasoning-capable LLMs whose predictions would come with underlying rationales. New models like Cohere’s Command-R^12^ can provide sources of information and a relatively structured line of reasoning. Its ability to answer questions with explicit reasoning steps shows how LLMs could attain a form of reasoning capacity, a hallmark of human intelligence. It does so through a technique called Retrieval-Augmented Generation (RAG). When given a query, the system first searches an external corpus for the most relevant documents, then generates a response conditioned on these retrieved facts. This design enables more faithful and verifiable outputs, addressing some explainability challenges inherent to purely generative LLMs. In the medical setting, Retrieval-Augmented Generation could enable models to back up any factual statements they present with citations to, e.g. medical textbooks, clinical guidance documents from standards bodies, or peer-reviewed scientific studies. Failures in retrieval quality or integration, however, can still introduce inaccuracies, underscoring the need for robust validation even in retrieval-augmented settings, and it still suffers from hallucinations and unfaithful reasoning^12–14^. Reasoning is, nonetheless, still an important capacity, in part, because it could help make LLMs more explainable by providing explicit rationales for predictions^14^. Work so far shows LLMs still have not attained the ability for complex reasoning, albeit having emerging characteristics. Instead of examples of true reasoning, the generated rationales could be reasoning-like steps drawing on the models’ vast training information^14^. A path forward is to test the models on more complex tasks and against more benchmarks in healthcare itself. Trying to make LLMs more explainable, thus, would both help test their reasoning capacities as well as make them more reliable tools for revolutionising healthcare.

Focussing efforts on making LLMs more explainable in healthcare does not necessarily need to suffer from the classical challenge of explainability versus performance. Through recently proposed approaches like Tree-of-Thoughts (ToT)^15^ and Graph-of-Thoughts (GoT)^16^, LLMs’ internal reasoning can be unpacked and visualised outside of the black-box and enhance predictive performance. Tree-of-Thoughts represents LLM reasoning in multiple linear paths similar to Chain-of-Thoughts or, in the case of Graph-of-Thoughts, as a graph where the nodes are collections of data and information. In the graph example, information processed by the LLM can be visualised with logical connections as edges in a graph between packets of data, while also providing the significance of each part of the graph to the model prediction. Since information critical to the final decision is highlighted, these approaches provide added explainability in LLMs. Both of these methods allow the LLMs to handle more complex tasks by mimicking human thought processes of exploring multiple pathways to the solution or representing information in a dynamically associative visual format, such as a dynamic graph. The traditional trade-off between increased explainability and decreased performance for machine learning models, thus, does not necessarily hold for LLMs. Further work is needed on applying these methods and similar ones in healthcare and evaluating them accordingly.

### Leveraging LLMs for multimodal healthcare applications

XAI, a popular term for eXplainability methods in AI, have previously struggled to deal with common multimodal and longitudinal settings in healthcare. A medical decision is often made as a result of a compendium of information being aggregated together, from medical history, longitudinal data like vital signs and lab measurements, to imaging or -omics data^17^. Besides relying on tree ensembles and Shapley values^18^, popularly explored methods in clinical XAI, there have been limited comprehensive proposals for explainability with multimodal AI in healthcare^19^. The introduction of LLMs into interpretation and interactive frameworks in clinical workflows provides a potential new tool in these settings. A recent review in npj Digital Medicine has highlighted the power and the limited ability of LLMs like GPT-4V in providing suitable interpretation in multimodal clinical settings^19^. The paper proposes a hypothetical LLM-based orchestrator which can generate verbal explanations adapted to the user and environment. Achieving robust explainability across diverse clinical environments remains a challenge due to differences in data drift, context sensitivity, and the complex causality inherent in medical decision-making^20,21^. Further research in development, interpretability, and evaluation is needed to contribute to the development of multimodal and longitudinal LLMs for clinical workflow implementations.

### Beyond technical solutions: regulatory and collaborative frameworks

Making LLMs in healthcare more explainable has to rely, however, on more than just technical solutions. Firstly, rigorous internal and external validation in clinical applications of AI models is needed^22^. Evaluating the explainability of LLMs in healthcare requires task-specific, stakeholder-aware assessment strategies. Explainability validation means not only assessing whether an output is factually correct, but also whether the reasoning is transparent, understandable, and trustworthy to the intended user, clinician, patient, or regulator.

Within the explainability criteria of transparency, accountability, trustworthiness, and interpretability, we suggest some relevant evaluation strategies:

1. Human-grounded evaluations: usability studies, clinician feedback on faithfulness and clarity of LLM rationales.
2. Functional tests or Retrieval-Augmented Generation-like approaches: prompt-based probing to test whether explanations align with source data or guidelines.
3. Fidelity metrics: comparing generated explanations to reference rationales or guideline-based answers in benchmark datasets.

For example, a faithfulness metric might test whether an LLM’s explanation for recommending a specific antihypertensive drug aligns with known clinical guidelines (e.g., National Institute for Health and Care Excellence (NICE))^23^. Conducting evaluations has to rely on cross-disciplinary collaboration (legal, sociological, clinical) not only for meeting these explainability criteria, but also for co-designing the evaluation protocols themselves^24^. What is explainable to a machine learning expert may not be actionable or trustworthy for a nurse practitioner or a regulator. Domain experts need to be included in system development cycles, research partnerships, or advisory roles to facilitate the smooth integration of LLMs into existing clinical workflows^19^. LLMs, then, must be developed and evaluated with a multipronged approach. Future work should develop shared benchmarks and participatory evaluation pipelines for LLMs in healthcare.

### Regulatory challenges and the need for adaptive frameworks

In parallel to technical developments, there needs to be added attention to developing robust safeguards and regulatory frameworks for LLM applications in healthcare. Bodies like the United States Food and Drug Administration (FDA) have struggled with regulating existing deep learning applications, partially due to their adaptive nature as they can adjust their parameters or behaviour based on the input data or their performance on a specific task^24^. As we mentioned earlier, this is especially true for LLMs whose new iterations from different companies tend to be released relatively quickly, and regulatory frameworks will struggle to keep up. A case in point is the FDA regulation of adaptive AI in radiology, where AI/ML-based algorithms that change over their product lifecycle pose regulatory challenges, as modifications require similar approval processes, which may create burdens given their frequent adaptive nature^25^. Similar issues are anticipated with LLMs, where version drift and fine-tuning updates complicate regulatory oversight^25,26^. Additionally, AI diagnostic tools for diabetic retinopathy, for example, have been challenged because clinicians could not sufficiently explain why certain decisions were made, demonstrating how legal standards can halt deployment even when technical performance is satisfactory, a fate awaiting future LLM proposals in healthcare^27–29^. Some suggestions for effective regulation include obligations regarding transparency, risk management, nondiscrimination provisions, and content moderation rules^2,24,30^. There also needs to be “specialised tools that enable better assessment of LLMs in the contexts and settings in which they will be used […] developed from scratch or adapted from existing LLM evaluation tools”^31^. These cannot be fulfilled without targeted, dedicated, and robust auditing capacities both within companies as well as governing bodies like the FDA. As hinted at in ref. ^31^, solutions also need to come from the entrepreneurial space for real-time in-clinical context evaluations of LLMs. Partnership with patients and clinicians in the design of evaluation procedures and red-teaming is also necessary. Red-teaming refers to the deliberate probing of AI systems, often by interdisciplinary experts, to identify unsafe, biased, or misleading behaviours before real-world deployment. In healthcare, red-teaming can simulate edge-case patient scenarios, adversarial inputs, or ethically complex decision contexts to assess how an LLM justifies its output and whether its explanations remain faithful and safe. OpenAI and others have used red-teaming frameworks in similar domains, and extensions are essential for clinical safety validation^32^. Next steps need to be taken to include feedback from domain experts in clinical practice and clinical AI to maintain the safety and increase the transparency of LLMs. Only by doing so from both the corporate and regulatory realms can the technology be robustly regulated.

### A roadmap for responsible LLM integration in healthcare

**Fig. 1:**
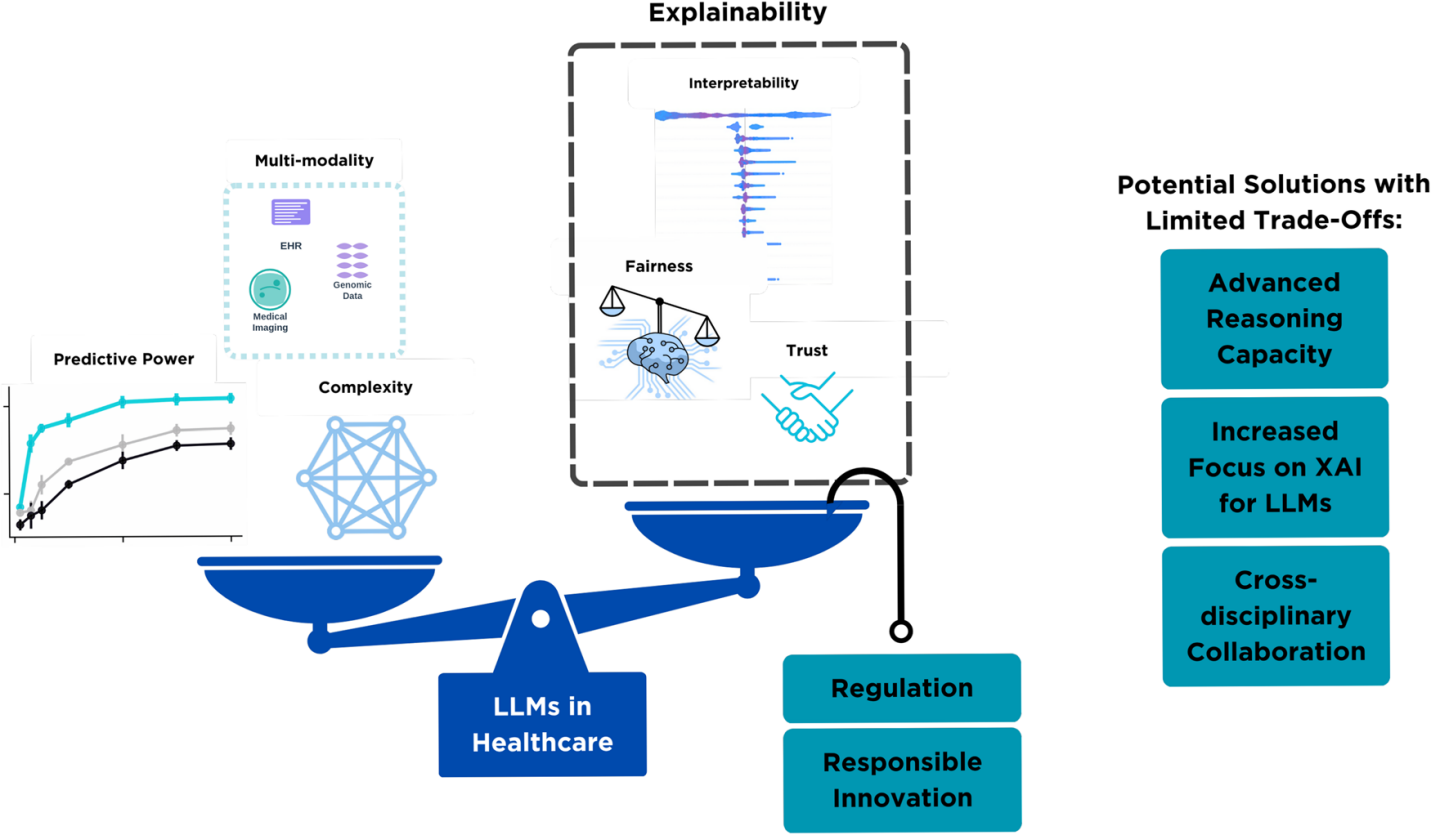
Balancing explainability requirements with model capabilities for LLMs in healthcare. The diagram shows a blue balance scale representing the trade-off between model development factors (left side) and explainability requirements (right side). The left side displays predictive power curves (blue and black lines), complexity (blue network), and multi-modality data types (purple EHR, teal medical imaging, purple genomics). The right side shows explainability components in a black dashed box: interpretability (blue bar chart), fairness (balance scales), and trust (handshake icon). Teal boxes on the right list potential solutions: Advanced Reasoning Capacity, Increased Focus on XAI for LLMs, and Cross-disciplinary Collaboration. The foundation shows LLMs in Healthcare (blue box) supported by Regulation and Responsible Innovation (teal boxes).

Overall, to help explain LLMs and implement them reliably, a combination of novel technical solutions, external validation, human-in-design approaches, and cross-disciplinary collaboration is needed, as highlighted in Fig. 1. A concrete vision for LLMs in healthcare involves their gradual integration into clinical workflows, guided by principles of risk management, explainability, and patient-centric design. LLMs could function as decision support partners during clinical consultations, providing clinicians with step-by-step rationales for differential diagnoses that are cross-referenced against up-to-date clinical guidelines (e.g., National Institute for Health and Care Excellence (NICE) guidelines)^33^. They could dynamically tailor medical explanations based on individual patient literacy levels, ensuring that outputs are accessible, culturally sensitive, and appropriately simplified without losing clinical accuracy. They could also orchestrate information from multimodal sources, electronic health records, imaging reports, and laboratory data, to generate summarised insights with transparent attribution to source data^34^. Integration of LLMs can follow a phased roadmap, aligning explainability requirements with clinical risk levels as shown in Table 1.

**Table 1:** A phased roadmap for responsible integration of large language models (LLMs) into healthcare workflows, aligning explainability requirements with clinical risk levels

Phase	Use Case	Risk Level	Explainability
Phase 1	Administrative tasks (documentation, discharge summaries)	Low	Basic traceability, example: Verily clinical note software^35^
Phase 2	Patient education materials, appointment summaries	Moderate	Literacy-adapted explanations, example: Babylon Health’s chatbot^36^
Phase 3	Clinical decision support (recommendations with clinician oversight)	High	Stepwise rationale + guideline cross-referencing, example: Google’s Med-PaLM 2 evaluated with expert review^37^
Phase 4	Autonomous triage, treatment suggestions (regulatory approval required)	Very High	Full transparency, auditability, and dynamic risk explanation

Adapted from best practices and emerging literature.

To take responsible advantage of the positive change which LLMs can provide in healthcare, it is essential to recognise that it is not a panacea. Human expertise and domain knowledge remain indispensable in providing care to patients. What we have learned from previous attempts at explaining AI is that we must involve contributions from across areas like ethics, law, clinical practice, and human-centred design. It will require innovative cross-disciplinary collaboration if we are to keep up with LLM progress in domains like healthcare, where trust, interpretability, and fairness are as important or more than predictive performance.
